# Leishmanicidal activity of saponins isolated from the leaves of *Eclipta prostrata* and *Gymnema sylvestre*

**DOI:** 10.4103/0253-7613.48891

**Published:** 2009-02

**Authors:** Venkatesan Gopiesh Khanna, Krishnan Kannabiran, Giulia Getti

**Affiliations:** School of Biotechnology, Chemical and Biomedical Engineering, VIT University, Vellore, Tamil Nadu, India; 1 School of Health and Bioscience, University of East London, Stratford Campus, Ramford Road, London, UK

**Keywords:** *Eclipta prostrata*, *Gymnema sylvestre*, leishmania promastigotes, Leishmanicidal activity, saponins

## Abstract

**Objective:**

To evaluate the leishmanicidal activity of saponin, dasyscyphin C of *Eclipta prostrata* and sapogenin, gymnemagenol from *Gymnema sylvestre* leaves under *in vitro* conditions.

**Materials and Methods:**

Dasyscyphin C/Gymnemagenol were dissolved in phosphate buffered saline (PBS) and diluted with liquid medium to obtain concentrations ranging from 1000 to 15 *μ*g /ml. The leishmanicidal activity against *leishmanial parasites, Leishmania major, Leishmania aethiopica* and *Leishmania tropica* promastigotes was studied by the MTS assay.

**Result:**

The Dasyscyphin C isolated from *E. prostrata* showed good leishmanicidal activity at 1000*μ*g/ml concentration, with the IC_50_ value of 450*μ*g/ml against *L. major* promastigote and the percentage of parasitic death was 73; whereas, gymnemagenol of G. sylvestre showed only 52% parasitic death at 1000 *μ*g/ml concentration. The other *Leishmania species, L. aethiopica* and *L. tropica promastigotes*, were less sensitive to the saponins of *E. prostrata* and *G. sylvestre*.

**Conclusion:**

From this study, it can be concluded that the dasyscyphin C of *E. prostrata* has significant leishmanicidal activity against *L. major* promastigote.

## Introduction

Leishmaniasis is one of the leading causes of morbidity and mortality and it has been found to be a major global health problem, with 1.5 to 2 million humans affected by the disease annually. Nearly 350 million people in 88 countries are estimated to be threatened by the disease.[1] It is caused by *Leishmania,* a protozoa transmitted by *Phlebotomus sp.*, commonly called sand flies. There are more than thirty species of sand flies responsible for the spread of Leishmaniasis. The available chemotherapy still relies on pentavalent antimonials, amphotericin B or pentamidine. Besides, they are reported to be associated with numerous toxic side effects, on prolonged use.[2,3] Hence, a search for a new active compound with potential leishmanicidal property remains essential for the development of a new antileishmanial therapy. Extracts from medicinal plants are being widely tested for leishmanicidal activity.[4] Studies on the antileishmanial activity of isolated active compounds from medicinal plants are scanty.

*G. sylvestre* leaves are traditionally used as medicine for the control of diabetes mellitus and stomach ache. They are also often used as a diuretic agent.[5] The decoction of *E. prostrata* leaves has long been used orally to control jaundice.[6] Earlier reports from our laboratory revealed the antimicrobial activity of gymnemagenol and dasyscyphin C.[7]

In the present study, the leishmanicidal activity of dasyscyphin C and gymnemagenol was evaluated against four *Leishmania* promastigotes under *in vitro* conditions.

## Materials and Methods

### Plant material

*E. prostrata* (Asteraceae) and *G. sylvestre* (Asclepiadaceae) leaves were collected in December 2007, from Jawadi Hills, Eastern Ghats, Tamil Nadu, India. The Voucher specimens were prepared and deposited at the Herbarium section of the VIT University, Tamil Nadu, India. The leaves of *G. sylvestre* and *E. prostrata* were washed with distilled water, shade dried, powdered and stored in an air- tight container until further use.

### Isolation and Purification of saponin

The powdered sample was defatted by petroleum ether for 3 h at 40°C. After filtering the petroleum ether, the sample was extracted with methanol for 3 h, with mild heating. The methanol extract was concentrated and re-extracted with methanol and acetone (1: 5 v/v) as described by Yan *et al*.[8] The precipitate obtained was dried under vacuum, which turned to a whitish amorphous powder after complete drying. It was loaded on Merck silica gel-60 (230-400 mesh) column and eluted with chloroform-methanol-water (70: 30: 10), as described by Favel *et al*.[9] The first fraction collected was air dried at room temperature (28°C) and the residue obtained was treated as the pure saponin of *G. sylvestre* and *E. prostrata*. The purity of the saponin isolated was analyzed by thin layer chromatography, using chloroform and methanol (7 : 3) as solvent system.

### Source of Leishmania species

All the cultures of the promastigote forms of *Leishmania* were obtained from the London School of Hygiene and Tropical Medicine and Armauer Hansen Research Institute, Addis Ababa, Ethiopia.

### Assay of Leishmanicidal activity

Saponins were dissolved in phosphate buffered saline (PBS) and diluted with liquid medium, to obtain the concentrations ranging from 1000 to 15 *μ*g/ml. The parasites were distributed in 96 well plates (Nunc, UK) (1×10^6^ parasites/ml) in triplicate, and each experiment was repeated twice. The plates were incubated for 24 h, at 22°C or 26°C, depending on the species being tested. Inhibition of promastigote growth was determined by the MTS assay.[10] The percentage of growth inhibition was determined by comparing the treated groups with the untreated controls after 48 hours.

### MTS assay

A solution of 3-(4,5-dimethylthiazol-2-yl)-5-(3-carboxymethoxyphenyl) -2- (4-sulfophenyl)-2H-tetrazolium, inert salt (MTS) was prepared (2mg/ml in PBS 0.02 M, pH 7.2) and stored at −20°C. Phenazine methosulfate (PMS, 0.92 mg/ml) was similarly prepared in PBS (0.02 M, pH 7.2) and stored at −20°C. For the evaluation, 20 *μ*l of a MTS/PMS (5:1) mixture was added to each well containing 200 *μ*l of treated and/or untreated *Leishmania* cultures, seeded at an initial concentration of 10^6^ cells/ml. The entire plate was incubated at 37°C for 3 h. The absorbance was measured at 490 nm and the percentage of viability calculated as described by Ganguly *et al*.[10]

### Structural Elucidation

The purified saponins were subjected to structural elucidation by Ultraviolet-visible spectroscopy (TECHCOMP), Fourier transform - Infrared spectroscopy (THERMO NICOLET -330), ^1^H-Nuclear Magnetic Resonance spectroscopy (JEOL GSX-500), ^13^C-Nuclear Magnetic Resonance spectroscopy (JEOL GSX-500) and Mass spectroscopy (FINNIGAN MAT-8230). All the chemicals used for extraction and purification were of analytical grade, obtained from SRL, Mumbai, India.

## Results

The leishmanicidal activity of saponins dasyscyphin C and gymnemagenol isolated from *E. prostrata* and *G. sylvestre* was tested against three *Leishmania* species. The viable parasites were counted in the culture media, to calculate the percentage of parasitic death at different concentrations of dasyscyphin C and gymnemagenol used to test the leishmanicidal activity. The effect of the different concentrations (1000, 500, 250, 125, 62.5, 31.25 and 15.625 *μ*g/ml) of dasyscyphin C of *E. prostrata* and gymnemagenol of *G. sylvestre* on the viability of *L. major* is shown in Table 1. The IC_50_ value for the inhibition of the growth of *L. major* promastigote was calculated to be 450 and 965 *μ*g/ml respectively for dasyscyphin C and gymnemagenol.

**Table 1:** Effect of dasyscyphin C and gymnemagenol isolated from the leaves of *Gymnema sylvestre* and *Eclipta prostrata* on *Leishmania major* promastigote

Saponin/Sapogenin (μg/ml)	% Growth of *Leishmania major*	

	Dasyscyphin C	Gymnemagenol
1000	27.01±3.04	48.39±8.53
500	51.33±13.70	76.77±6.71
250	62.41±10.06	81.10±2.38
125	69.96±4.29	90.26±4.00
62.5	69.50±13.30	98.76±8.24
31.25	76.06±7.10	96.84±2.00
15.625	88.82±3.30	110.73±12.66
0 (control)	100	100

Values are mean ± S.D. of six experiments.

**Figure 1:**
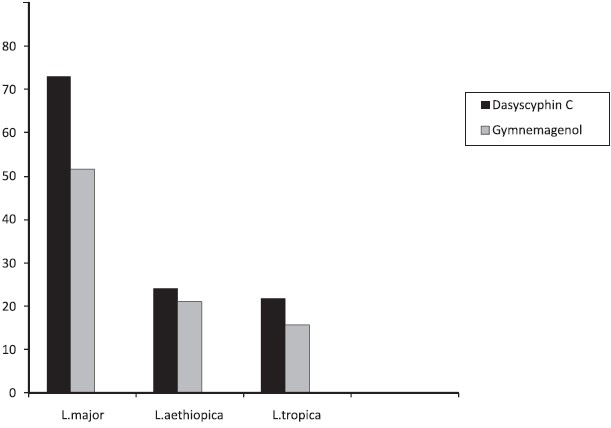
Effect of Dasyscyphin C and Gymnemagenol on *L. major, L. aethiopica* and *L. tropica* promastigotes. Parasitic death was calculated after 48 hours of exposure of Dasyscyphin C and Gymnemagenol (1000 μg/ml) of *E. prostrata* and *G.sylvestre*.

The dasyscyphin C and the gymnemagenol significantly inhibited (73 and 52% respectively) the growth of *L. major* promastigote at 1000 *μ*g/ml concentration [Figure 1]. The reduction in the growth of *L. aethiopica* and *L. tropica* promastigote forms was minimal (24 and 22% respectively) at 1000 *μ*g/ml concentration of dasyscyphin C. The gymnemagenol at 1000 *μ*g/ml concentration inhibited (22 and 16% respectively) minimally the growth of *L. aethiopica* and *L. tropica* promastigotes. The reduction in parasitic growth was maximal, when the parasitic culture was exposed to dasyscyphin C. Further, both dasyscyphin C and the gymnemagenol exhibited concentration dependent inhibition on the growth of *L. major.*

Dasyscyphin C. Jelly, ^1^H NMR (500 MHz in CDCl_3_), (OH) – 2.032 (m, 2H), 1-H -0.992 (t,2H), 2-H -0.889 (m,2H), 3-H -0.910 (t,2H), 5-H -1.316 (t,1H), 6-H -0.859 (t,2H), 7-H -0.876 (dd,2H), 9-H -1.301 (d,1H), 11-H -1.704 (t,2H), 12-H -5.197 (m,1H), 16-H -6.158 (s,1H), 18-H -1.629 (t,3H), 19,20-H -1.270 (s,6H), 21-H -2.310 (s,3H), 22-H -5.411 (m,2H), 22-H -5.411 (m,2H), 2′-H -3.683 (s,2H), 4′-H -4.069 (s,2H), 6′-H -1.149 (s,3H). ESI-m/z: 503 [M]^−^

Gymnemagenol. Crystalline needles, ^1^H NMR (500 MHz in CDCl_3)_,δ: 5.182 (9H,m,H-18,H-19,H-24); 5.104(1H,t,H-6); 4.060(3H,dd,H-11,H-12); 3.505(4H,S,H-16,H-17);2.790(1H,t,H-1); 2.313 (3H,m,H-5,H-4);2.291(2H,t,H-2); 2.084(1H,d,H-7);2.029(4H,m,H-20,H-25,H-26,H-27); 1.619 (3H, m,H-14,H-15); 1.275 (10H,m,H-3,H-8,H-9,H-10,H-13); 1.235(3H,s,H-23); 0.876 (6H,m, H-21,H-22). ESI- MS m/z: 474 [M]^+^

## Discussion

The leishmanicidal activity of dasyscyphin C and gymnemagenol, isolated from the medicinal plants *E. prostrata* and *G. sylvestre*, has been reported in this study. Saponins are the glycosides of triterpenoid or steroid aglycone as their backbone. The leishmanicidal activity of racemate type of compound derived from wood of *Cordia fragrantissima* has already been reported.[11] Leishmanicidal activity of a furostan-type of saponin from *Brunfelsia grandiflora* has been reported against *L. major* under *in vitro* conditions[12].

Recently, we reported that the isolated saponins from *G. sylvestre* and *E. prostrata* possess significant antibacterial and antifungal activity.[7] Further, these saponins have good cytotoxic activity on HeLa cells (unpublished data). However, the *in vitro* effects of these saponins on selected *Leishmania* promastigotes have not been reported till date. Thus, the present study was carried out to evaluate the leishmanicidal activity of these saponins on the growth of *Leishmania* promastigotes under *in vitro* conditions.

In our study, dasyscyphin C of *E. prostrata* was found to be more effective than gymnemagenol of *G. sylvestre* in inhibiting the growth of *L. major* promastigote. However, these saponins were not very effective against *L. aethiopica* and *L. tropica* promastigotes, even at higher concentrations tested.

Cardioquinol, isolated from the wood of *Cordia fragrantissima*, has been shown to contain very effective leishmanicidal activity at low concentrations (IC_50_ 81.2 *μ*g/ml).[11] As shown in Figure 1, *L. major* promastigote growth was significantly inhibited by the dasyscyphin C and gymnemagenol, with the concentrations of 500 and 1000 *μ*g/ml. This value indicates a significant inhibitory activity against *L. major* promastigote.

**Figure 2:**
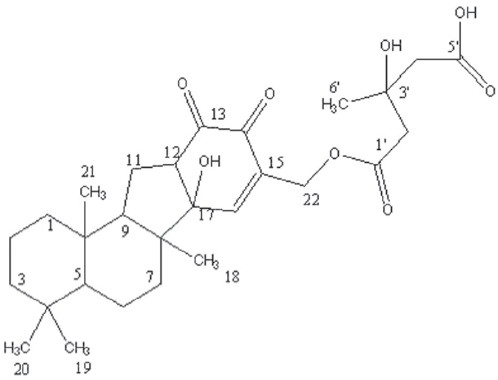
Structure of Dasyscyphin C

**Figure 3:**
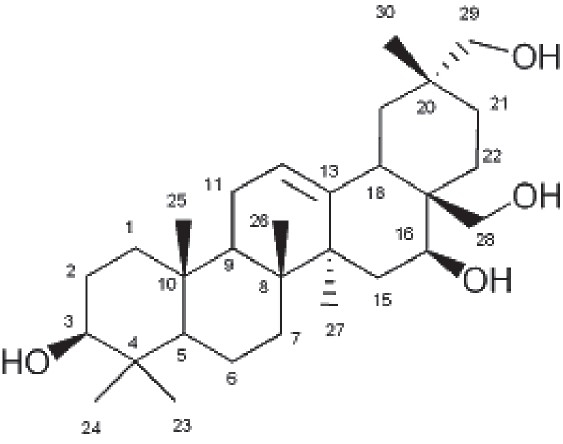
Structure of Gymnemagenol

Dasyscyphin C [Figure 2] and gymnemagenol [Figure 3] identity was proved by Thin layer chromatography, UV, FT-IR, ^1^H NMR and MS analysis. The presence of triterpenes in TLC plate was confirmed by Libermann-Burchard reaction and Carr-Price reagent. The UV spectrum showed the maximum absorption bands at 234, 238, 302 for dasyscyphin C and 223, 237, 274 for gymnemagenol, and the IR spectrum showed at 3435.80, 2921.82, 1635.05, 1245.75, 1050.66 for dasyscyphin C, and 3445.41, 2924.10, 1635.38, 1457.48 cm^−1^ for gymnemagenol. The FINNIGAN MAT 8230MS showed the [M] ^-^ion at m/z 503 with the base peak at m/z 208 for dasyscyphin C and for gymnemagenol showed the [M] ^+^ ion at m/z 474 with the base peak at m/z 251. The chemical shift assignments obtained for dasyscyphin C and gymnemagenol from ^1^H-NMR correspond to the molecular formula C_28_H_40_O_8_[13] and C_30_H_50_O_4_.[14]
